# Temporal and Spatial Regulation of Ezrin-Radixin-Moesin-Binding Phosphoprotein-50-kDa (EBP50) during Embryo Implantation in Mouse Uterus

**DOI:** 10.3390/ijms131216418

**Published:** 2012-12-03

**Authors:** Xing Li, Wang-Ming Xu, Tai-Lang Yin, Qing-Hong Zhao, Liang-Yu Peng, Jing Yang

**Affiliations:** Reproductive Medical Center, Renmin Hospital of Wuhan University, Wuhan 430060, Hubei, China; E-Mails: xingli1205@163.com (X.L.); mhlldc@yahoo.cn (W.-M.X.); xj_foreverfriends@126.com (T.-L.Y.); zhaoqinghong_0815@126.com (Q.-H.Z.); tmsygz2005@sina.com (L.-Y.P.)

**Keywords:** implantation, mouse, uterus, EBP50

## Abstract

Embryo implantation is a crucial process for successful pregnancy. To date, the mechanism of embryo implantation remains unclear. Ezrin-radixin-moesin-binding protein-50-kDa (EBP50) is a scaffold protein, which has been shown to play an important role in cancer development. Embryo implantation and cancer follow a similar progression. Thus, in this article, we utilized immunohistochemical staining and western blot analyses to examine the spatiotemporal expression and regulation of EBP50 both in the mouse uterus during embryo implantation as well as in other related models. We found that EBP50 was detected in epithelial cells in all of the groups used in our study. During the peri-implantation period, EBP50 mainly localized in apical membranes. At the implantation site (IS) on day 5 (D5) of pregnancy, EBP50 was mainly expressed in the nuclei of stroma cells, whereas from day 6 to day 8 (D6–D8) of pregnancy, the expression of EBP50 was noted in the cytoplasm of decidual cells. The expression of EBP50 was not significantly different in the pseudopregnant uterus and decreased in the uteri subjected to activation of delayed implantation. Artificial decidualization also decreased EBP50 expression. Thus, the expression levels and location were affected by active blastocysts and decidualization during the window of implantation.

## 1. Introduction

Embryo implantation is a reciprocal interaction between an implantation-competent blastocyst and a receptive uterus [1]. It is a crucial step for the successful establishment of mammalian pregnancy. There is a limited time period, known as the “window of implantation”, when the uterus and endometrium are receptive to the implanting embryo [2,3]. At the beginning of embryo implantation, the apical membrane of the luminal epithelium and the trophoblast of the adherent blastocyst interact with each other. Apical plasma membrane microvilli of uterine luminal epithelial cells change from long, thin, regular shapes during the receptive stage into irregular, flattened projections after the period of uterine receptivity [4]. Murphy *et al.*[5] suggested the term “the plasma membrane transformation”, which encapsulates changes in the plasma membrane of uterine epithelial cells during early pregnancy. However, in cancer progression, similar developmental procedures are reactivated [6]. Tumor progression and embryo implantation possess similar molecular mechanisms, such as epigenetic processes and dynamic regulation of cell migration and invasion [6,7].

EBP50 (ezrin-radixin-moesin-binding protein-50-kDa), a scaffold protein consisting of two tandem PDZ domains and a *C*-terminal ERM-binding region, interacts with a variety of membrane proteins and intracellular proteins, many of which have been shown to be related with cancer [8–10]. Until now, a major focus of research on EBP50 has been in the area of tumor progression. EBP50 has been shown to be enriched in polarized epithelial cells [8]. In the early stage of epithelial neoplasia development, epithelial polarity begins to be disrupted [11]. The loss of EBP50 from plasma membrane induces the plasma membrane transformation in epithelial polarized cells. Some studies show that EBP50 accumulates in the cytoplasm and nuclei of carcinoma cells, acting as an oncogenic agent [12], while other studies demonstrate that higher EBP50 expression could attenuate cancer cell proliferation [13–15]. EBP50 is also a well-characterized scaffold protein, highly expressed at the apical membrane of epithelial cells in many tissues, particularly in cells with numerous microvilli [8]. Many authors have validated that EBP50 is necessary for maintaining microvilli and that it can regulate the structure of microvilli [16,17].

Given the similarity between the processes of tumor progression and embryo implantation, we presume that EBP50 may play a role in implantation. Recently, Lecce *et al.*[18] showed that in rats, EBP50 occurs apically in uterine epithelial cells, and at the time of implantation, it was in response to blastocysts. However, up to now, the information on how EBP50 acts on the mouse uterus during embryo implantation was limited. Herein, we first examined the expression pattern of EBP50 in the mouse uterus during peri-implantation period. We also studied the effect of pseudopregnancy, artificial decidualization, delayed implantation and the activation of delayed implantation on the expression of EBP50. Finally, we observed the effect of steroid hormones on EBP50 expression.

## 2. Results and Discussion

### 2.1. Results

#### 2.1.1. Expression Pattern of EBP50 in Mouse Uterus during Early Pregnancy

**Figure 1:**
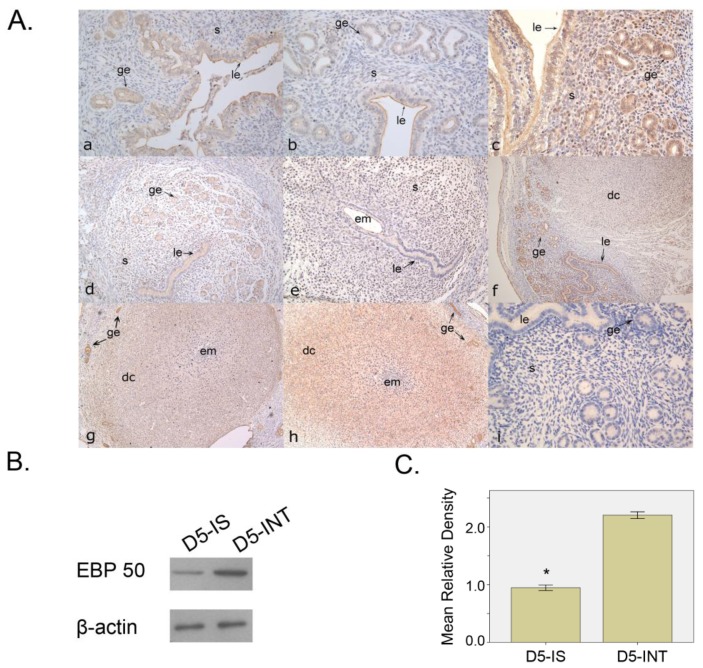
The localization and expression level of EBP50 in normal pregnancy. (**A**) The localization of uterine EBP50 during early pregnancy. **a**–**c**, Immunohistochemical analyses of EBP50 expression in the mouse uterus on days 2–4 of pregnancy; **d**, Immunohistochemical analyses of EBP50 expression in the inter-implantation sites (INT) on D5; **e**, Immunohistochemical analyses of EBP50 expression in the implantation sites (IS) on D5; **f**–**h**, Immunohistochemical analyses of EBP50 expression in the implantation sites (IS) on D6–8; **i**, Negative control conducted on a uterine section on D4, wherein the first antibody was replaced by PBS (Phosphate Buffered Saline). ge, glandular epithelium; le, luminal epithelium; s, stroma; em: embryo; dc, decidual cells; (**B**). Western blot analyses show the levels of uterine EBP50 expression on IS and INT of D5 of pregnancy; (**C**) The quantification of western blot findings. The mean relative density was calculated as the ratio of EBP50 to β-actin intensity. Asterisks indicate significant differences (*p* < 0.05).

On D2–D4 of pregnancy, the immunostaining of EBP50 was mainly detected in the apical membrane of the glandular and luminal epithelium, and a weak signal was also detected in subluminal stroma cells (Figure 1A(a–c)). On D5 of pregnancy, at the implantation site (IS), further away from the implantation site, we observed a stronger immunostaining signal mainly localized in the nuclei. Immunostaining was also observed in the glandular and luminal epithelium (Figure 1A(e)). At the inter-implantation site (INT), the immunostaining was similar to that on D2–D4 (Figure 1A(d)). On D6–D8, a positive signal was seen in decidual cells as well as in glandular and luminal epithelia (Figure 1A(f–h)); the EBP50 staining strength in the epithelium was markedly higher than that in the decidual cells. Western blot analyses showed that at the IS, the EBP50 expression level was lower than that at the INT (*p* < 0.05; Figure 1B,C).

#### 2.1.2. The Expression of EBP50 in Pseudopregnancy

**Figure 2:**
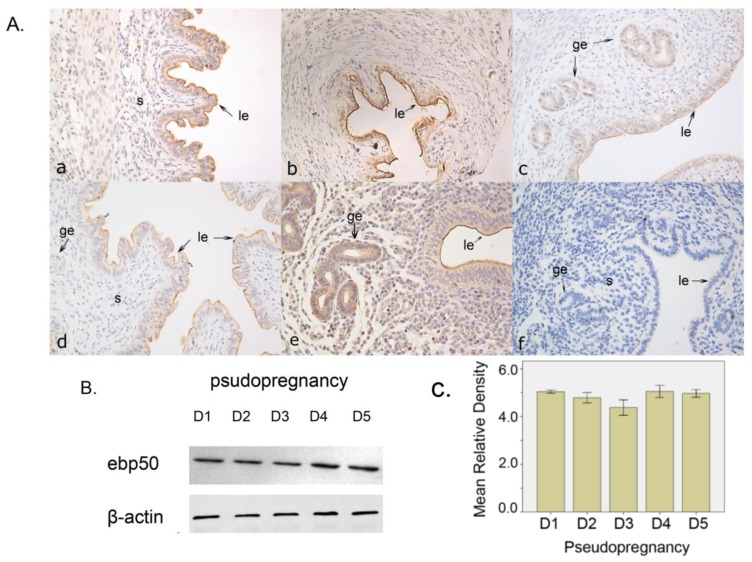
The localization and expression level of EBP50 in pseudopregnancy. (**A**) The localization of uterine EBP50 on D1–D5 of pseudopregnancy. **a**–**e**, Immunohistochemical analyses of EBP50 expression in mouse uterus on D1–D5 of pregnancy. **f**, Negative control conducted on a uterine section on D3 of pseudopregnancy, wherein the first antibody was replaced by PBS. ge, glandular epithelium; le, luminal epithelium; s, stroma; em: embryo; dc, decidual cells; (**B**) The levels of uterine EBP50 expression on D1–D5 of pseudopregnancy as detected using western blot analyses; (**C**) The quantification of western blot findings. The mean relative density was calculated as the ratio of EBP50 to β-actin intensity.

We utilized pseudopregnancy model to detect whether EBP50 expression was dependent on the presence of embryos. Immunohistochemical analyses showed that the EBP50 signal was mainly found in the uterine glandular and luminal epithelia during D1–D5 of pseudopregnancy. On D5 of the pseudopregnancy, a weak subluminal stroma signal could be detected (Figure 2A(a–e)). The expression level of EBP50 as detected by Western blotting was not significantly different in the uterus during D1–D5 of pseudopregnancy (Figure 2B,C).

#### 2.1.3. The Expression of EBP50 in Delayed and Activated Implantation

**Figure 3:**
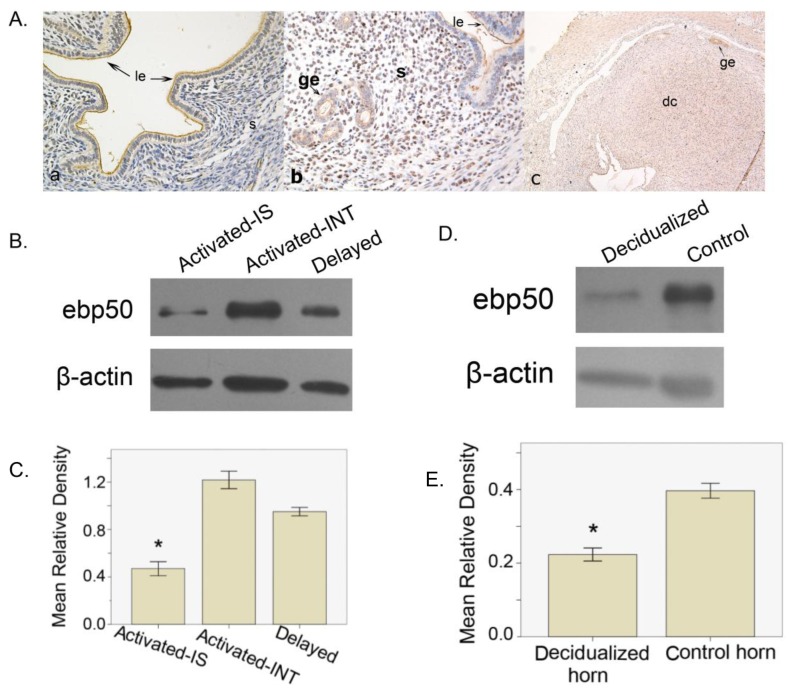
The localization and expression level of EBP50 in delayed, activated and decidualization models. (**A**) The localization of EBP50 in delayed, activated and decidualization models. **a**, delayed implantation. **b**, activation of delayed implantation. **c**, artificial decidualization. ge, glandular epithelium; le, luminal epithelium; s, stroma; em: embryo; dc, decidual cells; (**B**) Western blot analyses show mouse uterine EBP50 expression in the activated implantation sites (IS), the inter-implantation sites (INT) and in delayed implantation; (**C**) The quantification of western blot findings; (**D**) Western blot analyses show mouse uterine EBP50 expression in the decidualized horn and the control horn; (**E**) The quantification of western blot findings. The mean relative density was calculated as the ratio of EBP50 to β-actin intensity. Asterisks indicate significant differences (*p* < 0.05).

To test whether EBP50 expression was dependent on the embryo implantation status, a delayed implantation model was used. Immunohistochemical analyses showed that during delayed implantation, the main signal appeared in the uterine glandular and luminal epithelia, and a weak staining was observed in the subluminal stroma cells (Figure 3A(a)). After implantation was activated by estrogen treatment and the embryos had implanted, EBP50 expression was found in the nuclei of stroma cells as well as the glandular and luminal epithelia (Figure 3A(b)), which resembled the expression on IS of D5. From Western blot analyses, we observed that after activated implantation, the activated implantation sites had lower levels of EBP50 expression compared with that at the implantation sites (*p* < 0.05; Figure 3B,C).

#### 2.1.4. The Expression of EBP50 in Experimentally Induced Decidualization

Here, we use a model of experimentally induced decidualization to test whether EBP50 expression was regulated by decidualization. In the oil-infused uterus, strong signals were detected in the luminal and glandular epithelia, and a relatively weaker staining was observed in the induced decidual cells (Figure 3A(c)) The expression level of EBP50 in the decidualized uterus was clearly lower than that in the nonstimulated uterus on D8 of pseudopregnancy (*p* < 0.05; Figure 3D,E).

#### 2.1.5. The Expression of EBP50 in the Ovariectomized Mouse Uterus

**Figure 4:**
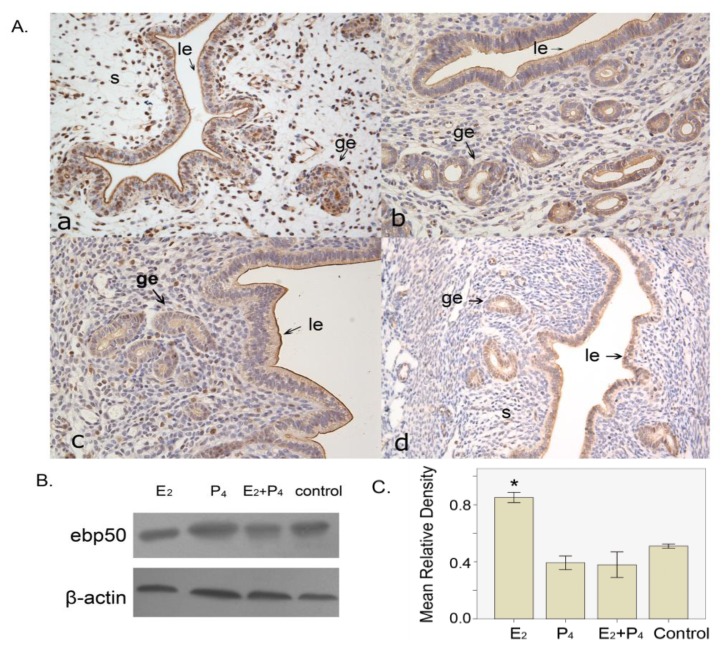
The localization and expression level of EBP50 in the steroid hormone treated models. (**A**) The localization of EBP50 in the steroid hormone(s)-treated uteri. **a**, estradiol-17β-treated uteri; **b**, progesterone-treated uteri; **c**, estradiol-17β plus progesterone treatment; **d**, the control group receiving vehicle only. ge, glandular epithelium; le, luminal epithelium; s, stroma; em: embryo; dc, decidual cells; (**B**) Western blot analyses show the mouse uterine EBP50 expression in the four groups; (**C**) The quantification of western blot findings. The mean relative density was calculated as the ratio of EBP50 to β-actin intensity. Asterisks indicate significant differences (*p* < 0.05).

EBP50 expression was only seen in the luminal and glandular epithelia of the ovariectomized mouse uterus, without any steroid hormone treatment (Figure 4A(d)). In the estrogen-treated group, in addition to the apical staining of epithelial cells, we also detected staining in the stroma cell nuclei (Figure 4A(a)). Furthermore, in the progesterone-treated group and in the estrogen plus progesterone-treated group, EBP50 expression was primarily present in the luminal and glandular epithelia, the staining was darker in the apical part compared with that in the cytoplasm, and there was also sporadic staining in stroma cells (Figure 4A(b,c)). Western blot analyses showed that the estrogen-treated group had higher levels of EBP50 protein expression than the other groups (*p* <0.05; Figure 4B,C).

### 2.2. Discussion

EBP50 was first identified in the rabbit renal brush border, and it was named NHE-RF (Na^+^-H^+^ exchanger regulatory factor) because of its cofactor action on NHE (Na^+^-H^+^ exchanger) type 3 [19]. Up to now, many studies have focused on EBP50, which is widely distributed and is particularly rich in tissues with polarized epithelia with numerous ezrin-radirin-moesin (ERM) family members, such as the liver, kidney, small intestine and placenta [20]. In addition to acting as a scaffold protein linking transmembrane proteins to various cytoskeletal proteins, EBP50 is localized in the plasma membrane and the cytoplasm of epithelial cells [21]. Hence, it can be deduced that EBP50 may be expressed in the apical plasma membrane of polarized epithelial cells. In this study, our results showed that during the peri-implantation period, the immunostaining of EBP50 in the uteri was mainly localized in the apical plasma membrane of the luminal and glandular epithelia, and the cytoplasm of epithelial cells had relatively weak immunostaining. We also observed sporadic staining among stroma cells. This expression profile is consistent with previous immunohistochemical findings [22] that a proliferative endometrium had strong EBP50 expression in the cytoplasm and membrane of the glandular epithelium as well as in scattered stromal cells. On D5, at the IS, the staining of EBP50 was mainly localized in the nuclei of surrounding stroma cells, while at the INT, the expression of EBP50 was still observed in epithelial cells. On D6–D8, when the primary decidual zone (PDZ) and secondary decidual zone (SDZ) had formed, the cytoplasm of decidual cells in the SDZ was stained, with the immunostaining intensity being weaker compared with that in glandular epithelial cells around the decidualization region. Previous work [23] has shown the binding of ERM proteins (ezrin, radixin, moesin) to EBP50 and the linking of F-actin to plasma membrane proteins, which are expressed in the luminal and glandular epithelia as well as stroma cells, during the early stage of pregnancy. Our Western blot analyses revealed that the level of EBP50 expression was lower in the IS than that in the INT. Compared with the uterine glands in the implantation sites, the inter-implantation site glands ablated more slowly without decidualization [24]. Thus, we supposed that the higher number and the stronger staining of uterine glands might explain the higher EBP50 expression in INT.

We have used pseudopregnancy as well as delayed and activated implantation models to explore whether the presence of blastocysts could change the mice uteri EBP50 expression pattern and level. The immunohistochemical staining of EBP50 from D1 to D5 of pseudopregnancy was analogous to that in D2–D4 of normal pregnancy. In the delayed implantation model, the EBP50 expression signal emerged from a similar area as that of the INT of mice uteri. After implantation was activated by estrogen, EBP50 expression was observed in the nuclei of stroma cells, similar to the IS in D5. These findings suggest that the appearance of blastocysts may change the location of EBP50.

Decidualization is a prerequisite for successful implantation; the decidual cells support embryo growth and regulate trophoblast invasion [25,26]. During decidualization, uterine stroma cells start to proliferate and differentiate into decidual cells, which also undergo extensive remodeling to control embryo invasion and accommodate the growing embryo. Boratko *et al.*[21] had reported that EBP50 is present in the nuclear and perinuclear regions in interphase cells. However, EBP50 redistributes to the cytoplasmic region during the prophase of mitosis. The findings of Boratko *et al.*[21] along with the proliferation of decidual cells could explain our model of artificial decidualization, wherein we observed that EBP50 localized in decidual cells and was mainly expressed in the cytoplasm.

In this study, the estrogen-treated mice had a higher EBP50 protein level than the other groups. The location of EBP50 in the four groups suggested that EBP50 expression in epithelial cells was independent of the steroid hormone. However, the presence of steroid hormone could influence the staining on stroma cells. This result coincided with those of other studies that support the contribution of estrogen to EBP50 expression. Fouassier *et al.*[27] indicated that in biliary epithelial cells, estrogens contributing to the proliferative response could regulate the distribution of EBP50. It was also previously reported that EBP50 is redistributed to the cytoplasm and nucleus of proliferative cells in estrogen stimulated-tissues, such as endometrium and breast tumors [28,29]. Our results showed that the association of estrogen with EBP50 expression is a positive signal in cell nuclei, and this is similar to EBP50 expression on D5 implantation that requires estrogen for the activation of blastocyst implantation.

EBP50 was first postulated as a mitogenic factor [30], and its diverse intracellular roles have been proposed later, such as an oncogene [12,28] or tumor suppressor [8–10,31]. Till now, the actual role of EBP50 in individual tissues remains obscure. Based on several different findings, a hypothesis has been proposed, which holds that different localizations of EBP50 are related to its different functions. EBP50 may have a tumor suppressive function in the apical cell membrane, while its localization in the cytoplasm and/or nuclei was associated with its function as an oncogene [20]. As seen through earlier studies described in the Introduction, embryo implantation is a similar process to tumor progression. In this study, during the peri-implantation period and the early stage of pseudopregnancy, apical cell membrane staining was clearly more prominent than cytoplasmic staining. At the IS on D5, EBP50 may participate in the transformation of stroma cells to decidual cells, as suggested by its changed location into nuclei. In addition, a cytoplasmic staining pattern for EBP50 was noted during decidualization, when the decidual cells were undergoing proliferation and differentiation. Thus, we presume that EBP50 also contributes to the process of decidualization.

In our study, implanting blastocysts and endometrium did not initiate reciprocal dialogue in pseudopregnancy or delayed implantation mice models, whereas, in the activated implantation mice model the blastocysts implantation was initiated. Ovariectomized model and artificial decidualization model afforded us the ability to study uterine function divorced from the hypothalamo-pituitary-ovarian axis. Thus, we utilized these models to elucidate the important role of blastocysts in spatio-temporal changes of the EBP50 expression in the mice uterus during the peri-implantation period.

Collectively, we suppose that in the peri-implantation stage, EBP50 mainly locating in the apical cell membrane maintains the normal cells’ shape and function; after embryo attaching the uteri epithelia, EBP50 shifts in nuclei and participate in the transformation of stroma cells to decidual cells; when the decidual cells have formed, EBP50 moves in cytoplasm to promote proliferation and differentiation.

## 3. Experimental Section

### 3.1. Animals and Treatments

Sexually mature mice (Kunming White outbred strain, 6–8 weeks) were purchased from the Laboratory Animal Center of Wuhan University (Wuhan, China). The mice were housed in a temperature- and humidity-controlled room with a 14/10 h light/dark cycle. All animal procedures were approved by the Institutional Animal Care and Use Committee of Wuhan University.

The female mice were caged overnight with fertile males or with vasectomy males of the same strain to induce normal pregnancy or pseudopregnancy. The presence of a vaginal plug or sperm was considered to be day 1 of pregnancy (D1) or pseudopregnancy (D1 of pseudopregnancy). In normal pregnant mice, uteri were collected from D1–D4, and the uteri together with embryo were collected from D5–D8. Whole uteri were collected from D1–D5 of pseudopregnancy. There were at least three mice in each group.

Artificial decidualization was induced by intraluminally infusing 25 μL of sesame oil (Sigma-Aldrich Inc., St. Louis, MO, USA) into one uterine horn on D4 of pseudopregnancy, whereas the contralateral uninjected horn served as a control. Uteri were collected on D8 of pseudopregnancy.

Our steroid hormone treatment models are described herein. Twelve normal sexually mature virginal mice were ovariectomized, and following a two-week recovery, steroid hormone treatments were initiated. The ovariectomized mice were given estradiol-17β (100 ng/0.1 mL/mouse), progesterone (1 mg/0.1 mL/mouse), or a combination of estradiol-17β and progesterone. The control mice received vehicle only (0.1 mL/mouse). All steroids were dissolved in sesame oil and injected subcutaneously (sc). Mice uteri were collected 24 h after the injection of vehicle.

To obtain the delayed and activated implantation models, six mice were divided into two groups, ovariectomized on the morning of D4 and maintained with daily injections of progesterone (1 mg/mouse in 0.1 mL of sesame oil, sc) from D5 to D6. On the morning of D7, one group was given subcutaneous injection of progesterone (1 mg/mouse in 0.1 mL of sesame oil, sc) and estradiol-17β (25 ng/mouse in 0.1 mL of sesame oil, sc) to induce blastocyst activation. During the same time, the other group received progesterone only. The mice were sacrificed to collect uteri 24 h following the estrogen treatment. Delayed implantation was confirmed by flushing blastocysts from one horn of the uterus.

Each collected tissue was divided into two parts. One part was first kept in liquid nitrogen and later stored at −80 °C for protein extraction. The other part was fixed in 4% paraformaldehyde (PFA) solution (Sigma-Aldrich Inc., St. Louis, MO, USA) for immunohistochemical analysis. The implantation sites of the activated uterus were identified through intravenous injection of 0.1 mL of 1% Chicago blue dye (Sigma-Aldrich Inc., St. Louis, MO, USA). The implantation sites and the inter-implantation sites were collected separately.

### 3.2. Immunohistochemical Staining

Mouse uteri were immediately cut into small pieces, fixed in 4% PFA solution, and embedded in paraffin. Four-micrometer sections were cut, deparaffinized, and rehydrated. The procedure followed the protocol described by Yu *et al.*[32], wherein the first antibody was replaced by the anti-mouse EBP50 (ab3452, Abcam, UK) at a dilution of 1:500.

### 3.3. Western Blotting

The uteri were grinded in Tissue Grinders, and lysed by a liquid mixture containing 0.2 mM phenylmethylsulfonylfluoride (PMSF) and 0.1 mM dithiothreitol (DTT). The nuclei were removed by centrifugation at 12,000× *g* at 4 °C for 15 min, and the supernatant was collected, aliquoted and stored at −20 °C until they were used. The protein samples (50 μg) were resuspended in the sample buffer (Beyotime, Jiangsu, China) and separated on 12% SDS-polyacrylamide gels. The protein samples were transferred to a nitrocellulose membrane by electrotransfer for 1.5 h. After being soaked in blocking buffer (5% Skim milk powder) for 2 h, the membranes were incubated with Anti-EBP50 antibody (1:1000 dilution) overnight at 4 °C. After three washes in TBST for 10 min, the membranes were incubated with matched HRP-linked secondary antibodies (1:5000) (Boster Bio-Technology Co., Ltd, Wuhan, China) for 1 hour, followed by three washes in TBST for 15 min. The signals were developed with an enhanced chemiluminescence (ECL; Beyotime, Jiangsu, China) reagent. The experiments were repeated three times.

### 3.4. Statistical Analysis

The statistical software, Statistical analysis SPSS 13.0, was used for data analysis. Data were calculated as the mean ± SEM. One-way analysis was used for the comparison of means between multiple groups. For comparing the means of independent samples, a *t*-test was used. Differences were considered to be significant at *p* < 0.05.

## 4. Conclusions

In conclusion, we found that EBP50 could be detected differentially in the mouse uterus during the early pregnancy period. The results gained from our models of delayed implantation, pseudopregnancy and artificial decidualization imply a significant role for implanting embryos and decidualization in the spatio-temporal changes of EBP50 expression in the uterus during the window of implantation. This expression of EBP50 was regulated by estrogen.

Overall, these findings will bring us a better understanding of the role of EBP 50 during the peri-implantation period and provide a foundation for further investigation on mechanisms of embryo implantation.
